# Insights into How Longicorn Beetle Larvae Determine the Timing of Metamorphosis: Starvation-Induced Mechanism Revisited

**DOI:** 10.1371/journal.pone.0158831

**Published:** 2016-07-07

**Authors:** Keisuke Nagamine, Yukio Ishikawa, Sugihiko Hoshizaki

**Affiliations:** Laboratory of Applied Entomology, Department of Agricultural and Environmental Biology, Graduate School of Agricultural and Life Sciences, The University of Tokyo, Tokyo, Japan; CNRS, FRANCE

## Abstract

Larvae of holometabolous insects must determine the timing of their metamorphosis. How they determine this timing has only been studied in detail for a few insect species. In a few species of Coleoptera, starvation is known to be a cue for metamorphosis, leading to the formation of smaller adults (starvation-induced pupation, SiP). We investigated the occurrence of SiP in the beetle *Psacothea hilaris*. When *P*. *hilaris* larvae were starved late in the feeding phase of the last (5th) instar, they exhibited typical SiP characterized by constancy of the time from food deprivation to pupation (TTP) irrespective of the body weight upon food deprivation or the length of prior feeding. In contrast, when larvae were starved early in the feeding phase, TTP decreased by roughly 1 day as the feeding became 1 day longer. The change in the response to starvation was estimated to occur on day 5.9 in the last instar. A series of refeeding experiments suggested that whereas SiP occurred readily in the larvae starved in the late feeding phase, activation of SiP was suspended until day 5.9 in the larvae starved early in the feeding phase. When *P*. *hilaris* larvae were fed continuously, they eventually ceased feeding spontaneously and pupated. The time length between spontaneous cessation of feeding and pupation was approximately equal to the TTP in SiP. This suggests that the same mechanism was activated by food deprivation in the late feeding phase and by spontaneous cessation of *ad libitum* feeding.

## Introduction

Metamorphosis is a type of molting through which an immature insect transforms into its adult form [1, 2]. Under optimal conditions for larval growth, holometabolous insects generally undergo a species-specific number of larval molts and eventually metamorphose into adults via the pupal stage. Last instar larvae must decide when to initiate the processes leading to metamorphosis; however, our knowledge on how they determine the timing of metamorphosis is limited to a few species of insects [3].

**Fig 1:**
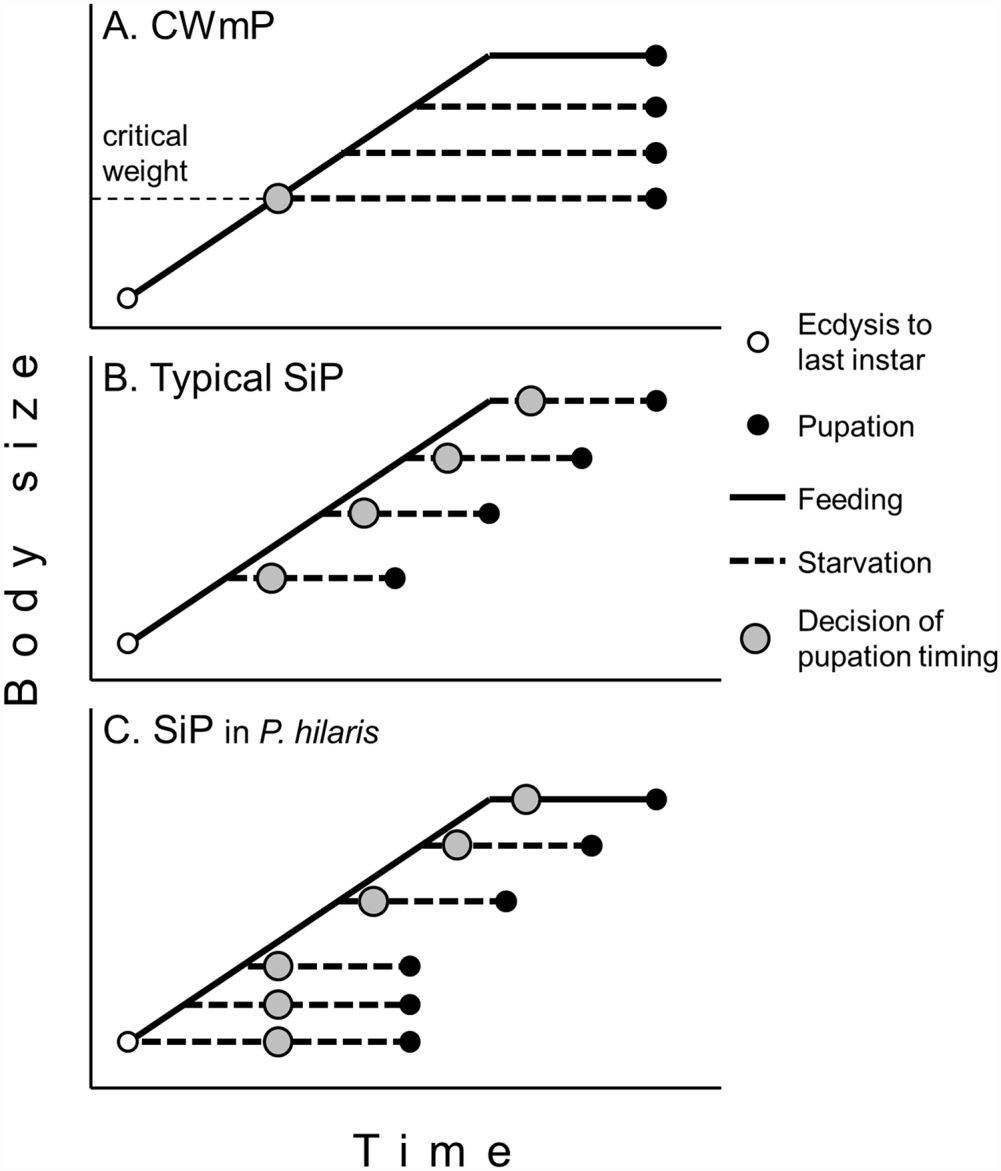
Models showing the timing of the decision to pupate in the last instar larvae of insects. A) Critical weight-mediated pupation (CWmP). B) Typical starvation-induced pupation (SiP). Modified from Nijhout (2008) [15].

The timing of and cue for larval decision toward metamorphic molting have been intensively studied in the tobacco hornworm *Manduca sexta* (Lepidoptera: Sphingidae) [3, 4]. The key signal for initiating metamorphosis is secretion of prothoracicotropic hormone (PTTH) from the brain, which stimulates secretion of ecdysone from the prothoracic glands. Last instar larvae of *M*. *sexta* cease feeding in response to a small surge of ecdysteroid, and subsequently pupate via wandering and prepupal stages. In the last instar, however, removal of hemolymph juvenile hormone (JH) is a prerequisite for the onset of metamorphosis in *M*. *sexta* because JH inhibits the secretion of PTTH from the brain [5, 6]. Processes to remove JH, i.e., cessation of JH synthesis and clearing of hemolymph JH by JH esterase, are initiated at the time when the last instar larvae of *M*. *sexta* attain a critical weight (CW) [5, 7]. JH is cleared from the hemolymph within an approximately constant time after the attainment of CW, which drives larvae to pupate. Since CW is attained in the middle of the feeding phase, food deprivation after a certain period of feeding does not affect the timing of pupation: the starved larvae become small pupae at the predetermined time point (Fig 1A). This mechanism to determine the onset of metamorphosis is referred to as critical weight-mediated pupation. Critical weight-mediated pupation has also been found in another moth, *Trichoplusia ni* (Lepidoptera: Noctuidae) [8], and the fruit fly, *Drosophila melanogaster* (Diptera: Drosophilidae) [9, 10], although critical weight-mediated pupation in *D*. *melanogaster* may not be identical to that in *M*. *sexta* [11].

Another mechanism to determine the onset of metamorphosis, starvation-induced pupation (SiP), has been found in Coleoptera (Fig 1B). Shafiei et al. [12] found that starvation of a larva of the dung beetle *Onthophagus taurus* (Coleoptera: Scarabaeidae) during the feeding phase in the last instar induces precocious pupation, provided that the larva has attained a minimum viable weight. The time between the start of starvation and the occurrence of pupation was constant regardless of the larval body weight upon starvation or the length of prior feeding, indicating that in *O*. *taurus* larvae, the decision to pupate is made a constant time after the start of starvation regardless of its timing in the feeding phase (Fig 1B). SiP is considered as a bailout mechanism that operates when an animal’s dietary environment deteriorates [3]. SiP has also been reported for two other beetles: fungus beetle *Dacne picta* (Erotylidae) [13] and blister beetle *Epicauta gorhami* (Meloidae) [14].

As described above, the mechanism to determine the timing of pupation appears to differ with the lineage of insects; however, the insect species studied to date are very limited in number and taxonomy [15]. The yellow-spotted longicorn beetle *Psacothea hilaris* (Coleoptera: Cerambycidae) is a pest of mulberry and fig trees [16]. The native range of *P*. *hilaris* is eastern Asia, but they have recently invaded Europe [16–18]. Their larvae infest branches of live host trees, and the beetles damage leaves. Adults of *P*. *hilaris* in the field show a large variation in body size, which affects mating behavior [19]. Two types of *P*. *hilaris* that differ in morphology, ecology, and physiology, i.e., west-Japan type and east-Japan type, are known to inhabit western and eastern parts of the main islands of Japan, respectively [20]. The control of larval development in *P*. *hilaris* has been intensively studied using the west-Japan type [21–23]. Under long-day conditions in the laboratory, west-Japan type *P*. *hilaris* larvae feeding on an artificial diet *ad libitum* pupate from the 4th or 5th instar [21, 23]. When the larvae are starved upon ecdysis into 5th instar, they precociously develop into pupae that are smaller than the counterpart raised under continuously-fed conditions [22]. A similar starvation-induced earlier pupation also occurs in 4th instar larvae [23, 24]. These findings suggest the presence of a SiP mechanism in *P*. *hilaris*.

The primary aim of the present study was to investigate whether SiP occurs in *P*. *hilaris*. We observed the occurrence of pupation in response to food deprivation at various timings in the 5th-instar larvae of *P*. *hilaris*. Unexpectedly, *P*. *hilaris* was found to exhibit an atypical SiP system: SiP occurred only in larvae starved in the late feeding phase of the instar. Based on our findings, we discuss conservative and nonconservative aspects of the SiP systems in Coleoptera, and also discuss the mechanism to determine the timing of pupation under continuously-fed conditions.

## Materials and Methods

### Ethics statement

Our study did not involve collection of endangered species. The species we collected, *P*. *hilaris*, is a pest of mulberry and fig trees. We obtained permission to collect this pest from Fukuchiyama City Hall.

### Insects

A laboratory colony of *P*. *hilaris* was established from about 50 adults of west-Japan type beetles collected at Fukuchiyama City, Kyoto, Japan, in July 2011. The maximum width of the elytra of these adults, which was used as a representative of body size, was measured using a Vernier caliper. The methods used to maintain stock cultures, e.g., rearing of adults, collection of eggs, and rearing of larvae, were previously described [23]. The 5th-instar larvae used for experiments in this study were obtained as follows. Larvae were individually reared from hatching on a piece of artificial diet (Silkmate ^™^ until the 3rd instar, thereafter replaced with Insecta^™^ LF; Norsan Corp., Yokohama, Japan) in Petri dishes (5.5 cm in diameter) under conditions of 25±1°C and short day (12 h light and 12 h dark; 12L:12D). The artificial diet was renewed every 3–5 days. In the later part of the 4th instar, the body weight of larvae and the occurrence of ecdysis were checked every 1–3 days. Larval ecdysis was recognized by the presence of the head capsule exuvia and/or coarse surface of thorax characteristic to a newly ecdysed larva. Upon molting into the 5th instar, larvae were weighed and transferred to a long day condition (15L: 9D) to expedite pupation [25]. Most larvae were expected to pupate in the next (5th) molt by this photoperiodic change [25].

### Continuous feeding

**Fig 2:**
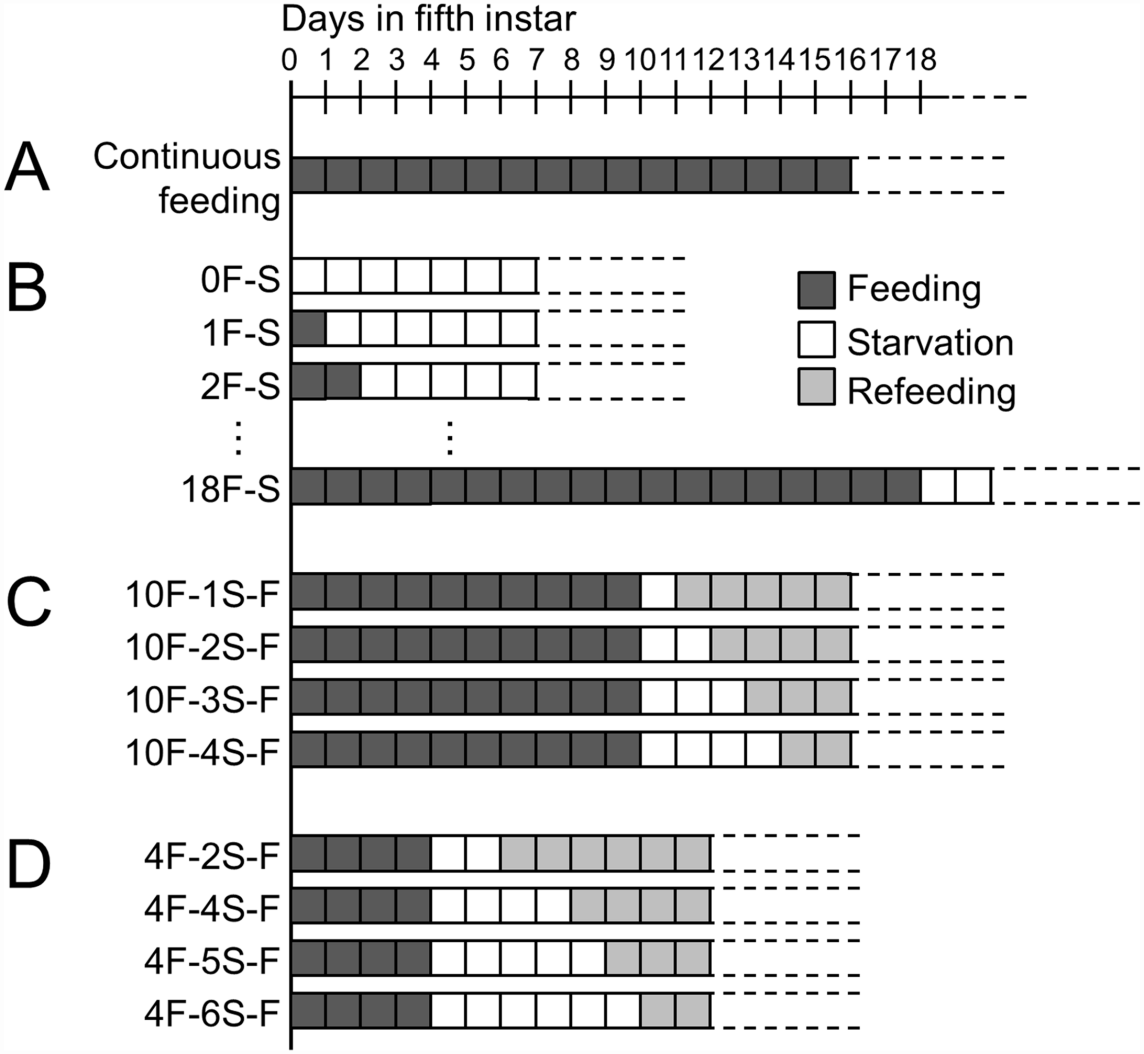
Designs of feeding/starvation experiments. A) Continuous feeding. B) Feeding/starvation. C and D) Starvation/refeeding experiments.

Newly ecdysed 5th-instar larvae were obtained as described above, and the larvae were allowed to feed on the artificial diet *ad libitum* until pupation (Fig 2A). The body weight of larvae was measured every 1–2 days to determine the peak weight. The day a larva reached peak weight was regarded as the day the larva ceased feeding spontaneously. The occurrence of gut purge was recorded for each larva. Transition of a larva to a prepupa was noted when the larva did not lift its head in response to physical stimulus. Pupal weight was measured 0–2 days after pupation. Pupae were individually maintained in Petri dishes and checked every 2–3 days for adult emergence. The maximum width of the elytra of adults was measured using a Vernier caliper.

### Starvation

Newly ecdysed 5th-instar larvae were fed for 0–18 days, and then deprived of food (Fig 2B). In this series of experiments, 4F-S, for example, indicates that 5th-instar larvae were fed for the first 4 days and then starved. The body weight at food deprivation, which represents peak weight, was recorded. The larvae were observed every 1–3 days for up to 40 days in the 5th instar. When the feeding period became longer than 8 days, some larvae spontaneously ceased feeding to prepare for pupation. To identify these insects, body weight of larvae that had fed for more than 8 days was monitored every 1–2 days, and larvae that lost weight were excluded from subsequent analyses. Larvae that did not pupate nor ecdyse to the 6th instar during the observation period were recorded as ‘not ecdysed’. Larvae that died during the observation were excluded from most analyses. Gut purge and transition to a prepupa were noted as described above. The body size of adults was measured as described above.

### Refeeding after starvation

Newly ecdysed 5th-instar larvae were fed for 4 or 10 days, deprived of food for 1–6 days, and then refed (Fig 2C and 2D). In this series of experiments, 10F-2S-F, for example, indicates that larvae were fed for 10 days, starved for 2 days, and then fed again. The body weight of larvae at the start of 5th instar and the body weight at the time of food deprivation (peak weight) were recorded. The body weight of larvae was also measured upon refeeding and on the next day to detect the ingestion of diet. Any larvae that ceased feeding spontaneously before food deprivation were excluded from the experiments.

### Statistical analyses

R was used for statistical analyses, mostly together with R commander [26, 27].

## Results

### Effects of starvation on pupation

When newly ecdysed 5th-instar larvae were starved without any access to food, as many as 58% of them molted into pupae (Table 1), indicating that growth in the 5th instar is not an absolute prerequisite for successful pupation. When the larvae were fed for one day before starvation, the pupation rate increased to 69%. As the feeding was extended to 2 days or more, the pupation rate reached >85%, which was comparable to that of the larvae allowed to feed *ad libitum* (Table 1).

**Table 1:** Pupation rate at the next ecdysis of 5th instar *Psacothea hilaris* larvae grown under different feeding/starvation regimens *

	Continuously fed	0F-S	1F-S	2F-S	3F-S	4F-S	5F-S	Late- starved ^§^
Pupation rate (%)	85	58	69	90	86	90	86	90
(n)	(39)	(26)	(13)	(23)	(22)	(40)	(14)	(94)

* F and S indicate feeding and starvation, respectively.

1F-S, for example, indicates that newly ecdysed 5th instar larvae were fed for 1 day and then starved (See Fig 2A for details).

^§^ Late starved: data for 6F-S to 18F-S were combined.

**Fig 3:**
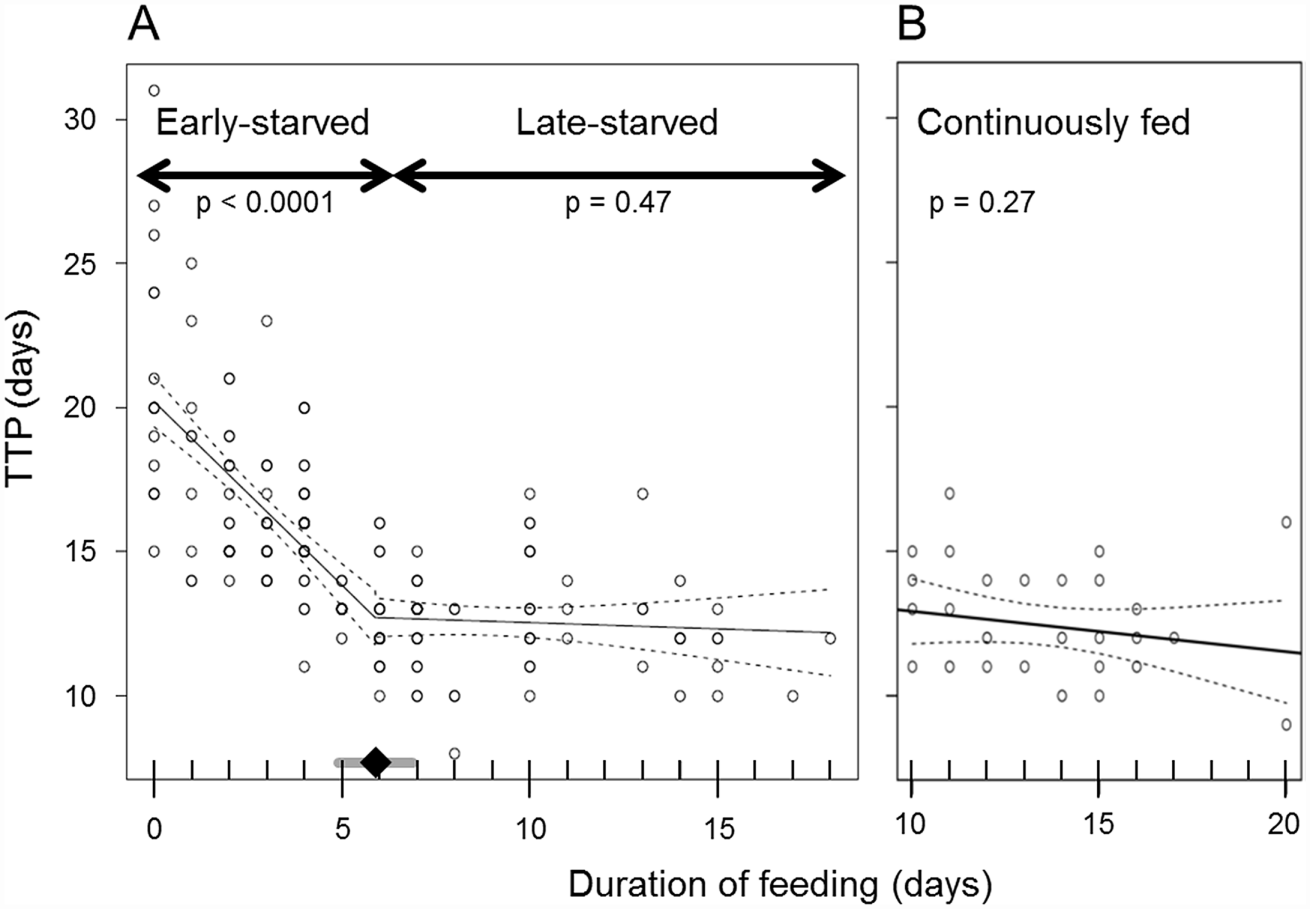
Relationship between the duration of prior feeding and the time from forced/spontaneous cessation of feeding to pupation (TTP). Larvae under continuously fed conditions were assumed to have spontaneously ceased feeding on the day the larvae exhibited peak weight. A) Results of feeding–starvation experiments. Thin line with a breakpoint represents the result of a bisegmental linear regression analysis, and accompanying broken curves indicate the 95% confidence limits of the regression. The black diamond and gray bar near abscissa indicate the break point estimate (day 5.9) and its 95% confidence interval, respectively. B) Results of continuous feeding experiments. The solid line and broken curves indicate the linear regression line and its 95% confidence limits, respectively.

The time from food deprivation to pupation (TTP) is expected to be constant regardless of the prior feeding duration if the larvae pupate via the typical SiP mechanism. In *P*. *hilaris*, TTP was not constant across the range of feeding period (Fig 3A). The response of larvae to starvation appeared to change before and after a certain time point. A bisegmental linear regression analysis [28, 29] showed a breakpoint on day 5.9. In the group of larvae fed for 0–5 days before starvation (the early-starved group), TTP was negatively correlated with the duration of feeding (Y = −1.3X + 20; adjusted r^2^ = 0.36, p < 0.001, n = 111). TTP decreased by roughly 1 day as the larvae were fed for 1 day longer. In the group of larvae fed for 6–18 days before starvation (late-starved group), TTP was nearly constant (12.6 days) irrespective of the duration of feeding (adjusted r^2^ = 0.00056, p = 0.47, n = 85; Y = −0.043X + 13).

In the group of larvae continuously fed throughout the 5th instar, TTP (here, time from spontaneous cessation of feeding to pupation) was not significantly correlated with the duration of feeding (adjusted r^2^ = 0.0078, p = 0.27, n = 3; Y = −0.14X + 14) (Fig 3B). The average TTP was 12.4 days, which was almost equal to that for the late-starved group (12.6 days).

Although the pupal durations in the early-starved, late-starved, and continuously-fed groups were not significantly different (p > 0.05 by ANOVA, F = 3.37), the 5th instar periods in the three groups were significantly different (ANOVA, F = 60.24, p < 0.001), in the order of early-starved < late-starved < continuously-fed (Tukey’s test, p < 0.05). The pupal weight was also significantly different among the three groups (ANOVA, F = 31.41, p < 0.001), and was in the order of early-starved < late-starved < continuously-fed (Tukey’s test, p < 0.05).

### Effects of body weight on determination of pupation

To examine whether the body weight of larvae affected determination of the timing of pupation in *P*. *hilaris*, larvae were first divided into 4 groups by their initial weight in the 5th instar: 200–249 mg, 250–299 mg, 300–349 mg, and 350–399 mg. Linear regression analysis of TTP to the duration of feeding was conducted for each of the early (< 6 days) and late (≥ 6 days) starved subgroups in each initial weight group. TTP was negatively correlated with the duration of feeding in the early-starved subgroup, and the regression line was nearly horizontal for the late-starved subgroup (S1 Fig). Next, larvae were divided into six groups by the peak weight in the 5th instar: 200–299 mg, 300–399 mg, 400–499 mg, 500–599 mg, 600–699 mg, and 700–799 mg. Regardless of the peak weight, TTP decreased as the duration of feeding increased in the early-starved subgroup, whereas TTP was not significantly correlated to the duration of feeding in the late-starved subgroup (S2 Fig). These results verified that body weight did not affect determination of the timing of pupation.

### Developmental stage affected by SiP

TTP can be divided into two parts by the transition to prepupae (Table 2). Whereas no significant difference was found in the duration of prepupa among the three feeding–starvation regimens (ANOVA, F = 2.30, p > 0.05), the time from food deprivation/spontaneous cessation of feeding to prepupa differed significantly (ANOVA, F = 39.48, p < 0.001). Thus, the difference in the TTP among the three regimens was attributable to the difference in the time spent to become a prepupa.

**Table 2:** Effects of starvation on the time (days) from food deprivation to prepupa and prepupal duration.

Regimen *	Food deprivation to prepupa ^§^		Prepupal duration ^§^	
	Mean (S.D.)	n	Mean (S.D.)	n
Continuously fed	12.4^a^ (1.9)	33	4.4^a^ (0.9)	33
Early-starved	16.6^b^ (3.3)	111	4.8^a^ (0.9)	25
Late-starved	12.6^a^ (1.7)	85	4.4^a^ (0.8)	34

* Early-starved: data for larvae fed for 0–5 days prior to starvation (0F-S to 5F-S in Fig 2A) were combined.

Late-starved: data for larvae fed for 6–18 days prior to starvation (6F-S to 18F-S in Fig 2A) were combined.

^§^ Means in the same column with the same letter are not significantly different (Tukey test, p < 0.05).

The occurrence of gut purge in the larvae reared under 4F-S, 10F-S and continuously-fed regimens was recorded (Table 3). The gut purge continued for two or more days. The larvae in 4F-S and 10F-S regimens started to purge their guts 5.3 days (95% confidence interval, 4.1–6.5 days) and 2.3 days (95% confidence interval, 1.6–2.9 days) after food deprivation, respectively. The larvae in the continuously-fed group started to purge their guts 2.4 days (95% confidence interval, 2.0–2.8 days) after spontaneous cessation of feeding, which was not significantly different from the time from food deprivation to gut purge in the 10F-S regimen (ANOVA, F = 0.17, p > 0.05).

**Table 3:** Effects of starvation on the time (days) from food deprivation to initiation of gut purge.

Regimen *	Mean (S.D.) ^§^	n
Continuously fed	2.4^a^ (1.2)	33
Early-starved	5.3^b^ (2.4)	15
Late-starved	2.3^a^ (1.3)	15

* See the footnote to Table 2.

^§^ Means with the same letter are not significantly different (Tukey test, p < 0.05).

### Size variation in adult beetles

**Fig 4:**
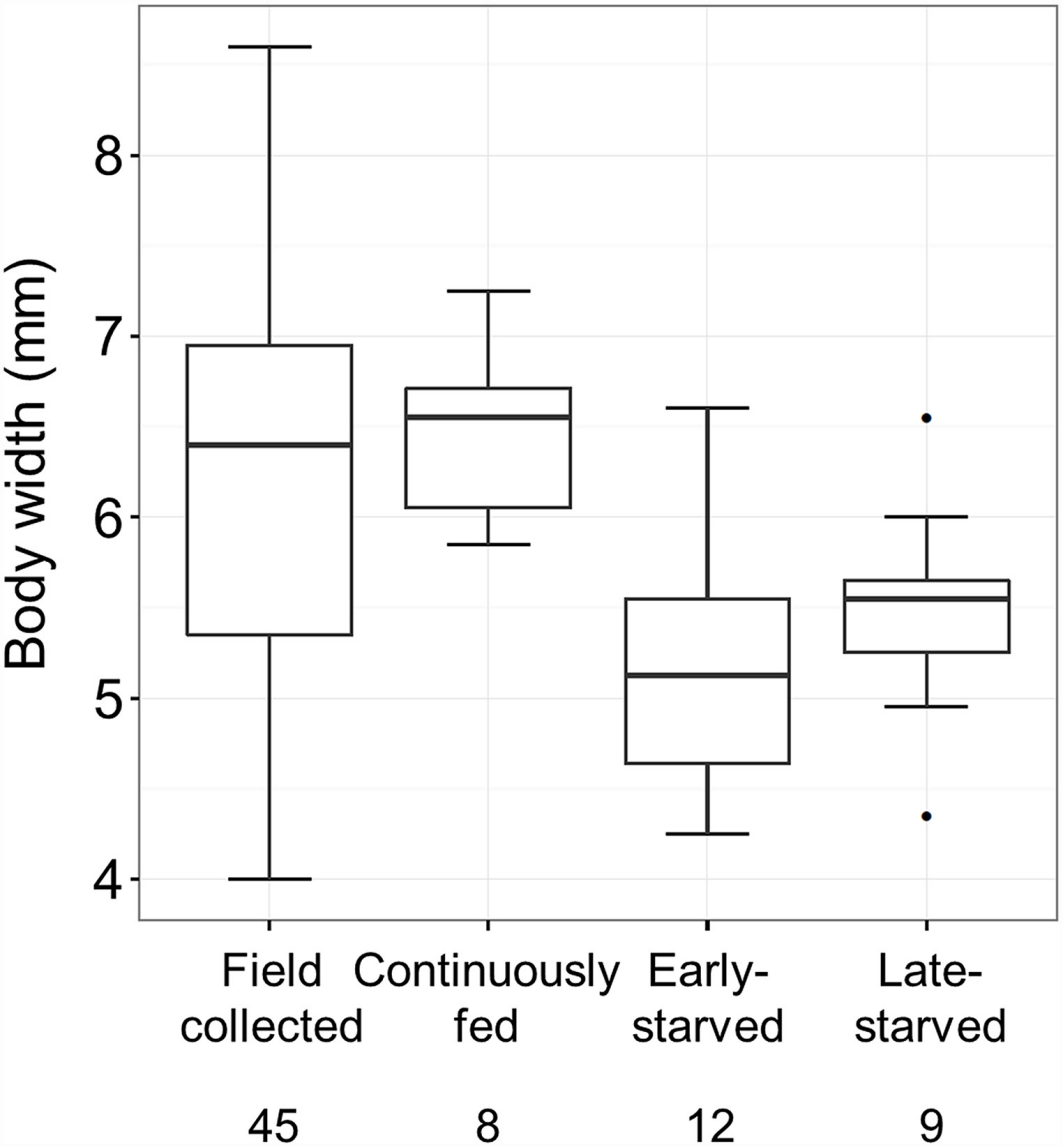
Adult body size of beetles collected in the field, those raised in the laboratory under continuously fed conditions, and those raised in the laboratory under starved conditions. The numbers in the bottom indicate sample sizes.

The body size of beetles emerging from the early-starved, late-starved, and continuously-fed groups was compared with that of beetles collected in the field (Fig 4). The variation in the body size of the field-collected beetles (4.0–8.6 mm) was so large that its range included the ranges of the beetles from the early-starved group (4.3–6.6 mm), late-starved group (4.4–6.6 mm), and continuously-fed group (5.9–7.3 mm). The median size of the field-collected beetles (6.4 mm) was close to that of the continuously-fed group (6.6 mm). Larvae of the early- and late-starved groups developed to beetles, the size of which corresponded to small beetles in the field.

### When late-starved larvae decided to pupate

**Fig 5:**
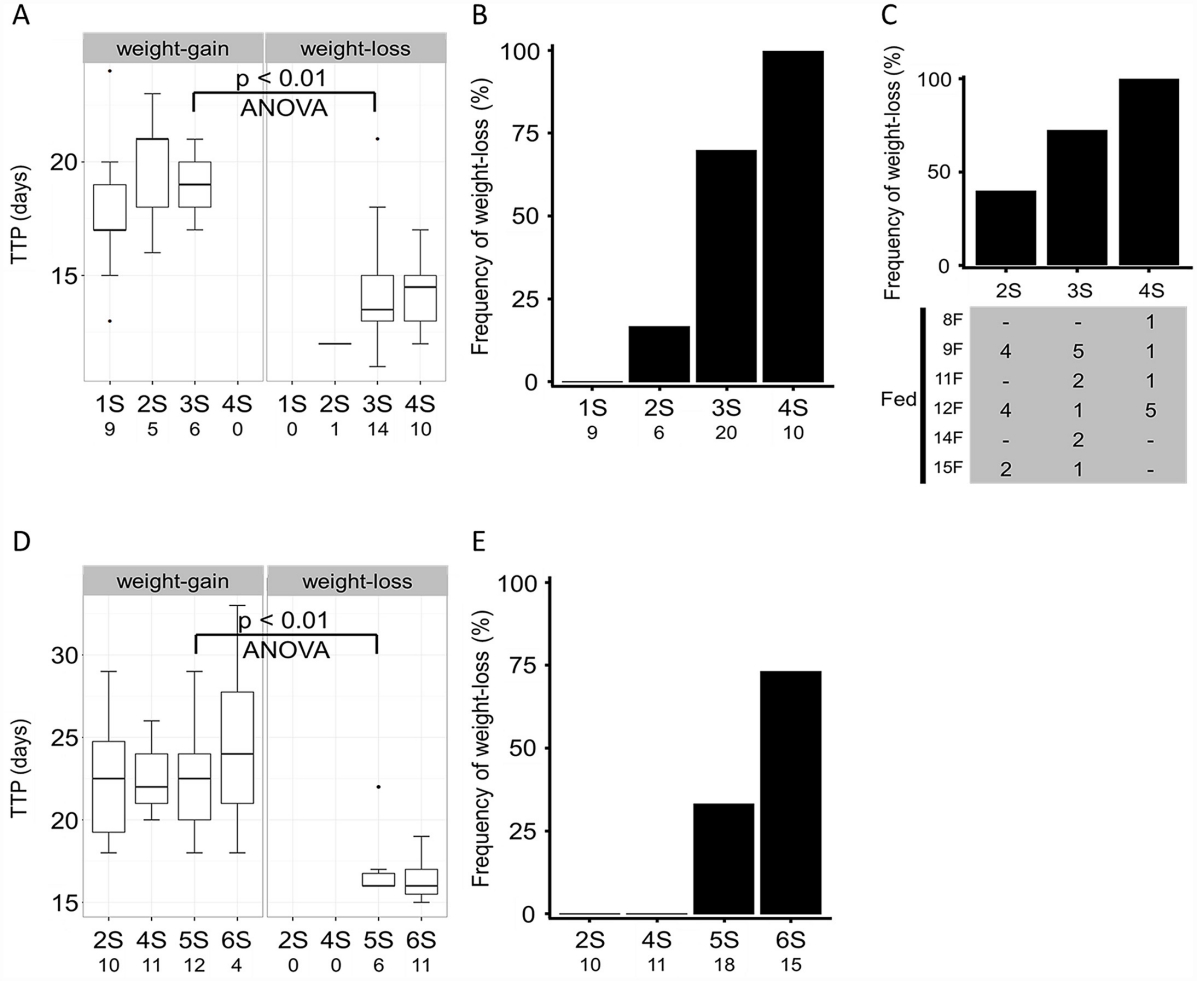
Effects of the duration of starvation on pupation timing. Newly ecdysed 5th instar larvae were fed for 10 days (A and B), 8–15 days (C), or 4 days (D and E). They were then starved for 1–4 days (A and B), 2–4 days (C), or 2–6 days (D and E), and subsequently fed again. Larvae that gained and those that lost body weight on the next day of refeeding were analyzed separately. A and D) Relationship between the duration of starvation and TTP. B, C, and E) Frequency of larvae that lost weight on the next day of refeeding. Numbers at the bottom of panels (A, B, D, and E) and in the gray box (C) indicate sample sizes.

Larvae that had decided to pupate were expected not to ingest any more food. We tested whether this also applies to starved larvae by refeeding them. First, we observed responses of larvae that had been fed for 10 days and starved for 1, 2, 3, or 4 days (Fig 2B). Larvae that gained/lost weight during the first day of refeeding were regarded to have/not to have ingested food. The ratio of animals pupated at the 5th ecdysis was >75% regardless of the duration of starvation (S2 Table). When the larvae starved for different durations were pooled, TTP in the weight-gain group (mean = 18.7, SD = 2.7, n = 20) was significantly longer than that in the weight-loss group (mean = 14.4, SD = 2.3, n = 25) (ANOVA, F = 33.78, p < 0.001) (Fig 5A). The mean TTP in the weight-loss group (14.4) was close to that in the larvae reared under the 10F-S regimen (mean = 13.4, SD = 2.1, n = 15) (ANOVA, F = 1.73, p > 0.05). In the 10F-3S-F regimen, TTP in the weight-gain group (mean = 19.0, SD = 1.6, n = 6) was significantly longer than that in the weight-loss group (mean = 14.6, SD = 2.7, n = 14) (ANOVA, F = 13.55, p < 0.01) (Fig 5A). These results indicate that larvae that had decided to pupate because of the starvation did not ingest food even if food became available afterward. Hereafter, larvae that lost weight during the first day of refeeding under late-starved conditions were considered to have determined to pupate during the starvation regimen.

The effects of the duration of starvation on the decision to pupate were analyzed next (Fig 5B). No larvae starved for one day determined to pupate. The number of larvae that had determined to pupate increased as the duration of starvation was extended, and all of the larvae starved for 4 days had determined to pupate. The duration of starvation necessary for 50% of larvae to determine pupation was estimated at 2.7 days (95% confidence interval, 2.1–3.0 days). Pupal weight was not significantly affected by the duration of starvation in either the weight-gain or weight-loss groups of larvae (S3 Table). The duration of starvation had no effect on the duration of pupal stage (S3 Table).

To see whether the tendency observed in the above starvation experiments, which used larvae fed for 10 days, is generally observed in larvae fed for ≥ 8 days, newly ecdysed 5th-instar larvae were fed for 8, 9, 11, 12, 14, or 15 days, starved for 2–4 days, and then refed (Fig 5C). The larvae fed for different durations were pooled for analysis. Forty percent, 73%, and 100% of larvae starved for 2, 3, and 4 days, respectively, did not gain weight (= did not ingest food), indicating that they had already determined to pupate at the time of refeeding. The duration of starvation required for 50% of larvae to decide to pupate was estimated to be 2.3 days (95% confidence interval, 1.0–2.8 days).

### When early-starved larvae decided to pupate

Newly ecdysed 5th-instar larvae were fed for 4 days, starved for 2, 4, 5 or 6 days, and then refed (Fig 2D). There was no significant difference in peak weight among the larvae starved for different durations (S4 Table). All of the larvae examined pupated at the 5th ecdysis (S4 Table). When the larvae starved for different durations were pooled, TTP in the weight-gain group (mean = 22.7, SD = 3.3, n = 37) was significantly longer than that in weight-loss group (mean = 16.6, SD = 1.7, n = 17) (ANOVA, F = 50.8, p < 0.001) (Fig 5D). The mean TTP in the weight-loss group (16.6) was close to that in the larvae reared under the 4F-S regimen (mean = 15.9, SD = 1.9, n = 36) (ANOVA, F = 2.19, p > 0.05). In the 4F-5S-F regimen, TTP in the weight-gain group (mean = 22.4, SD = 3.0, n = 12) was significantly longer than that in the weight-loss group (mean = 17.2, SD = 2.4, n = 6) (ANOVA, F = 14.03, p < 0.01) (Fig 5A). These results indicate that larvae that lost weight during the first day of refeeding had already determined to pupate. Analysis of the relationship between the frequency of weight-loss larvae and the duration of starvation showed that while none of the larvae starved for up to 4 days had determined to pupate, 33% and 73% of larvae starved for 5 and 6 days had determined to pupate, respectively (Fig 5E). The duration of starvation for 50% larvae to determine pupation was estimated at 5.4 days (95% confidence interval, 5.1–5.9 days). Duration of starvation had no effect on the duration of pupal stage (S5 Table). The pupal weight in both the weight-gain and weight-loss groups was not significantly different regardless of the duration of starvation (S3 Table).

## Discussion

### Modified SiP in *P*. *hilaris*

**Fig 6:**
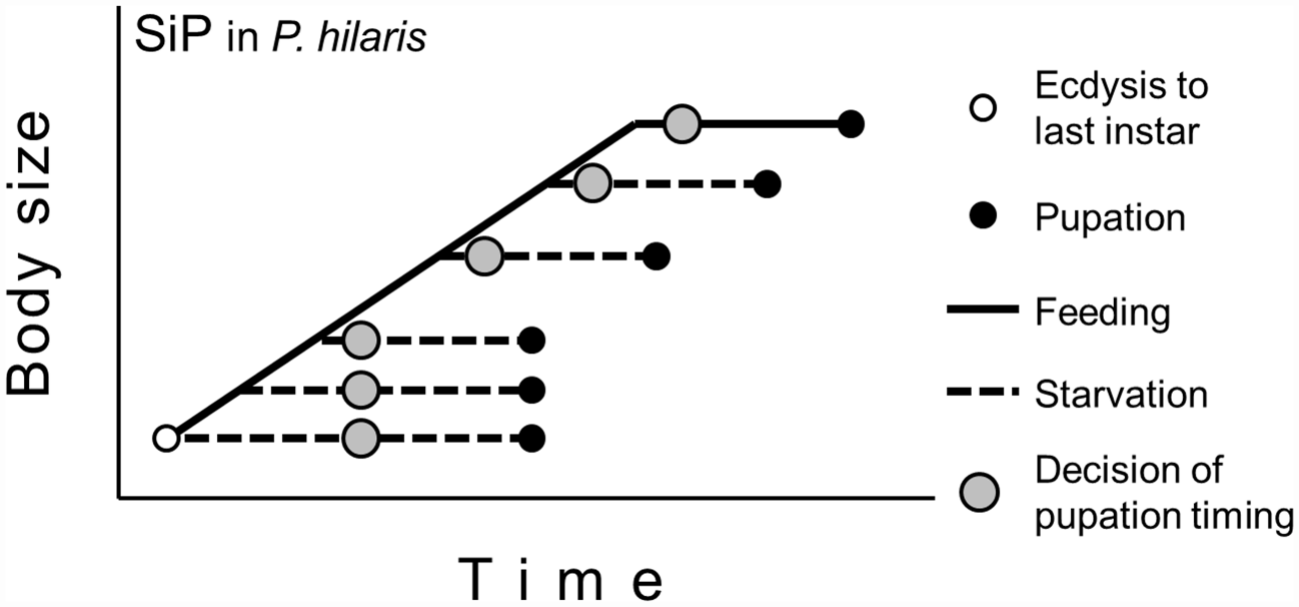
A model showing the timing of decision to pupate in the last instar larvae of *Psacothea hilaris*. A modified SiP in *P*. *hilaris*.

Our study clarified that last instar larvae of *P*. *hilaris* exhibit not a typical, but a modified SiP, as discussed next (Fig 6). Starvation experiments revealed that the feeding phase in the last (5th) instar of *P*. *hilaris* comprises two parts distinguished by the response to starvation. Larvae starved on day 6 or later pupated after a constant TTP regardless of their body size or the duration of prior feeding, indicating that a typical SiP occurred in the later part of the feeding phase. Refeeding experiments revealed that in larvae starved during the later part, the decision to pupate was made 2.7 days after food deprivation. This timing agrees with the initiation of gut purge in 10F-S larvae (Table 3). In contrast, for larvae starved in the early part of the feeding phase, TTP was not constant but became shorter by approximately 1 day as the feeding was extended for 1 day. The change in TTP was attributed to the change in the time from food deprivation to the transition to prepupa. Based on these results, we argue that last-instar larvae of *P*. *hilaris* starved during the early part of feeding phase suspended their decision of pupation until day 5.9. In other words, the SiP mechanism was nonfunctional until day 5.9 in the last instar.

Results of the refeeding experiments of early-starved larvae are explained as follows. When newly ecdysed last-instar larvae were starved after 4 days of feeding, SiP was suspended for 1.9 days until day 5.9 when the larvae became SiP-competent. The decision to pupate was made 2.7 days thereafter, i.e., on day 8.6. Refeeding experiments with the larvae starved for 2–6 days from day 4 (Fig 5E) revealed that the decision to pupate was made 5.3 days after food deprivation, i.e., on day 9.3 in the last instar. The two estimations of the date of decision making, 8.6 days and 9.3 days after food deprivation, were fairly close.

### Variants of SiP mechanism in Coleoptera

SiP has been reported for beetles belonging to three different families: Scarabaeidae, Erotylidae, and Meloidae. Our study found a variant of the SiP mechanism in *P*. *hilaris*, a species belonging to a different family, Cerambycidae. Since the above four families in Coleoptera are not phylogenetically close to each other [30], SiP mechanisms may prevail among various other lineages of Coleoptera. Our study also demonstrated the occurrence of variation in SiP. SiP in the last-instar larvae of *P*. *hilaris* is atypical in that it is nonfunctional until day 5.9. Body growth in the last larval instar is not a prerequisite for SiP in *P*. *hilaris* and *E*. *gorhami* [14, 22], whereas last instar larvae of the scarab beetle *O*. *taurus* and the erotylid beetle *D*. *picta* require a substantial body growth to become SiP-competent [12, 13].

### Why does *P*. *hilaris* larvae possess a SiP mechanism?

In the field, adults of *P*. *hilaris* show a large variation in the body size [19, the present study]. One of the causes of this size variation is the variation in the number of larval molts in this beetle: individuals that have undergone more molts tend to be larger [21]. We argue that SiP may be another cause of the size variation in the field. The starved 5th-instar larvae of *P*. *hilaris* developed into adults of smaller size than those fed continuously [22, the present study]. The body size of beetles emerging via SiP and that of beetles emerging under continuously-fed conditions were both within the body size variation seen in the field. The size of laboratory beetles that emerged via SiP corresponded to that of small beetles in the field. These findings suggest that SiP occurs in the field and is responsible for the large variation in the body size. The SiP mechanism has also been considered to be responsible for the large body size variations of *O*. *taurus* and *D*. *pict* in the field [12, 13].

To date, SiP has been found in species whose larval food often becomes exhausted during larval life. In *O*. *taurus*, female adults provide the offspring with brood balls made of manure, and no further food is available to the offspring [12, 31]. In *D*. *picta*, the larval food, mushrooms, becomes degraded in a short period, and it is often difficult for larvae to find additional food [13]. In *E*. *gorhami*, larvae are provided with a locust egg pod as the sole food source [14]. Under these dietary conditions, SiP is considered adaptive for the survival of these species [3]. In contrast, larvae of *P*. *hilaris* infest branches of live Moraceae plants, and thus their food should be sufficient in amount for larval growth and development. The quality of the larval food may deteriorate rapidly if the branch infested by *P*. *hilaris* larvae is broken by strong wind. Larvae in such branches may become starved and pupate by the SiP mechanism; however, such a situation would not occur often in the field. Therefore, the adaptive significance of SiP in *P*. *hilaris* is not clear. Possibly, SiP may have been inherited from ancestral species as an evolutionary constraint on the life history rather than an adaptation to their dietary environment.

### Pupation mechanism under continuously-fed conditions

When last instar larvae of insect species possessing a SiP mechanism are continuously fed, they spontaneously cease feeding and pupate [12, 14, 22]. In the present study, last instar larvae of *P*. *hilaris* fed continuously spontaneously ceased feeding on day 13.5 on average, during the late part of feeding phase, and pupated on day 26.0. Therefore, continuously-fed insects must use a mechanism other than SiP for determining the timing of their pupation. This mechanism remains unresolved, and is likely to be different from the critical weight-mediated pupation in *M*. *sexta*. Our study showed that TTP in the late-starved *P*. *hilaris* larvae and TTP in the larvae that spontaneously ceased feeding are not significantly different (Fig 3). A similar finding was reported for *O*. *taurus* [12]. In *P*. *hilaris*, continuously-fed larvae and those starved after 10 days of feeding purged the gut at similar timings after food deprivation and spontaneous cessation of feeding, respectively. Therefore, in the continuously-fed and late-starved conditions, the pupation should have been determined at an identical time point after the forced/spontaneous cessation of feeding. Considering these findings and the SiP mechanism together, we propose that spontaneous cessation of feeding determines the pupation timing in the last instar larvae of *P*. *hilaris* fed continuously: the same signal for metamorphosis, which is yet to be clarified, is considered to be released both when starved in the late part of feeding phase and after the spontaneous cessation of feeding. This hypothesis regards SiP not only as a bailout mechanism in the case of food exhaustion but also as a part of developmental processes leading to pupation under an optimal dietary environment. However, this hypothesis does not explain the mechanism that leads to the spontaneous cessation of feeding in last instar.

### Possible hormonal control of metamorphosis

Hormonal control of metamorphosis in the last instar larvae of *P*. *hilaris* is of interest. It is well accepted that a small surge of hemolymph ecdysteroid titer in the last instar induces gut purge and subsequent metamorphosis [32–34]. A small surge of ecdysteroid before the gut purge was also observed in *P*. *hilaris* [35]. The present study showed that the timing of the gut purge is close to that of the determination of pupation. Munyiri and Ishikawa [36] found that the small surge of ecdysteroids occurred earlier in the starved larvae than in the continuously-fed larvae. These findings lead us to speculate that starvation during the late feeding phase in last-instar *P*. *hilaris* affected the release of ecdysteroid for the initiation of metamorphosis. It is interesting to know the signal that is released in response to the cessation of feeding. In *D*. *melanogaster*, starvation leads to a decrease in the transcripts of insulin-like peptides [3]. Starvation-induced changes in the expression levels of insulin-like peptides may be involved in the SiP in beetles. In the last larval instar of *M*. *sexta*, nutrition-mediated growth of prothoracic gland is required for it to become competent to secrete ecdysteroids [37, 38]. This may not be the case in *P*. *hilaris* because the last instar larvae of *P*. *hilaris* pupate in response to starvation even if they are starved from the beginning of the instar.

## Supporting Information

S1 Fig The effects of initial weight in the 5th instar on the relationship between prior feeding duration and time from food deprivation to pupation (TTP). Samples of Fig 3A were divided into four groups (panels A–D) based on the initial weight in the 5th instar. Red and blue colors indicate the early- and late-starved groups, respectively. The 95% confidence intervals of the regression segments are indicated by shades.
(PDF)

S2 Fig The effects of peak weight in the 5th instar on the relationship between prior feeding duration and time from food deprivation to pupation (TTP). Samples of Fig 3A were divided into six groups (panels A–F) based on the peak weight in the 5th instar. Red and blue colors indicate the early- and late-starved groups, respectively. The 95% confidence intervals of the regression segments are indicated by shades.
(PDF)

S1 Table Initial weight, duration of 5th instar, pupal weight, and pupal duration of *Psacothea hilaris*, larvae of which were starved early or late in the 5th instar. (PDF)

S2 Table Results of refeeding experiments (late-starved). Weight at food deprivation and pupation rate in the 5th instar *P*. *hilaris* larvae fed for 10 days prior to starvation. (PDF)

S3 Table Results of refeeding experiments (late-starved). Initial weight at 5th instar, pupal weight in weight-gain and weight-loss groups, and pupal duration in *P*. *hilaris*, larvae of which were fed for 10 days prior to starvation. (PDF)

S4 Table Results of refeeding experiments (early-starved). Weight at food deprivation and pupation rate in the 5th instar *P*. *hilaris* larvae fed for 4 days prior to starvation. (PDF)

S5 Table Results of refeeding experiments (early-starved). Initial weight at 5th instar, pupal weight in weight-gain and weight-loss groups, and pupal duration in *P*. *hilaris*, larvae of which were fed for 4 days prior to starvation. (PDF)
