# Correction: Insights into How Longicorn Beetle Larvae Determine the Timing of Metamorphosis: Starvation-Induced Mechanism Revisited

**DOI:** 10.1371/journal.pone.0162213

**Published:** 2016-08-25

**Authors:** The PLOS ONE Staff

Fig 1C is incorrectly included in the image of Fig 1. Please see the correct Fig 1 here. The publisher apologizes for the error.

**Fig 1:**
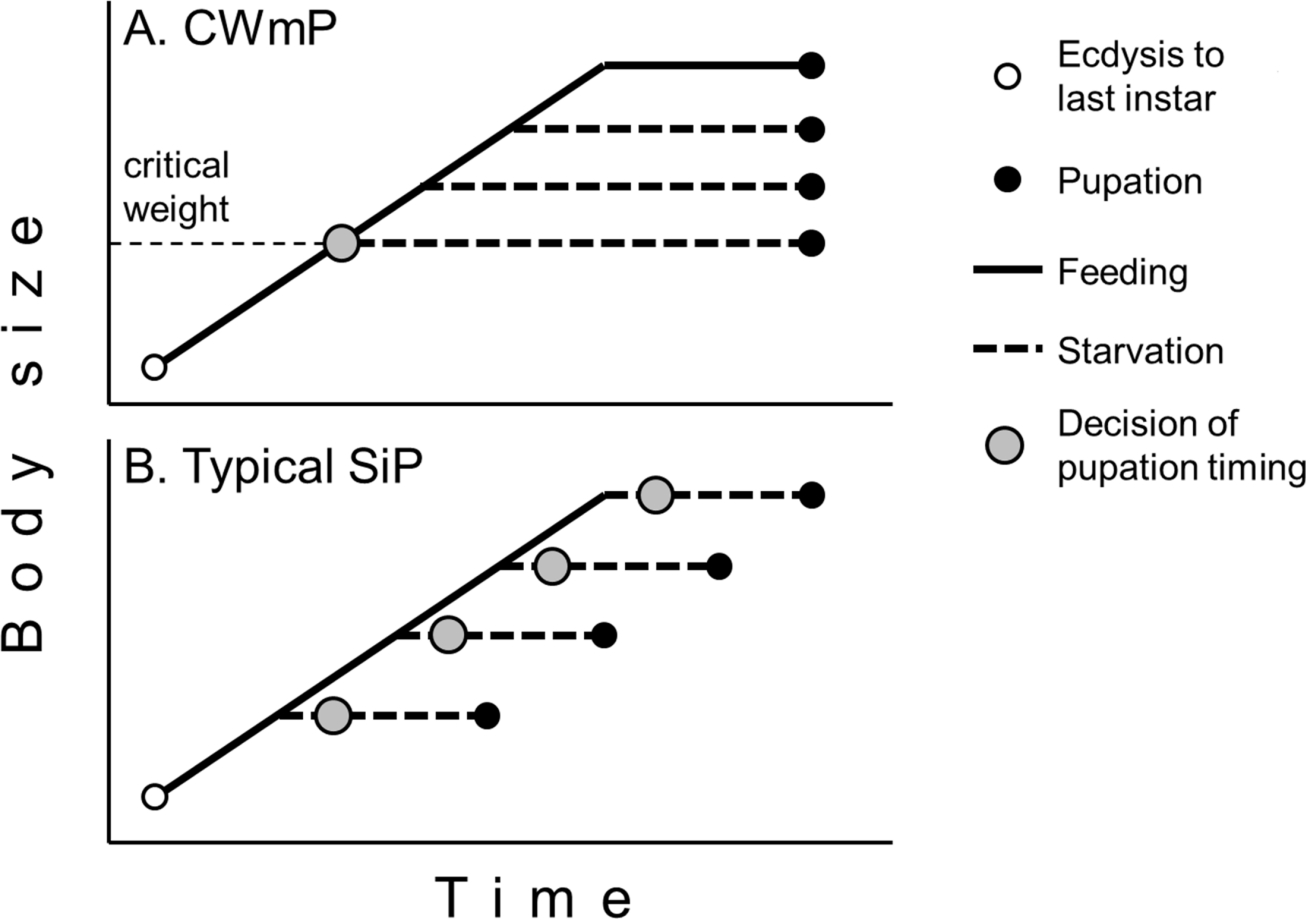
Models showing the timing of the decision to pupate in the last instar larvae of insects. A) Critical weight-mediated pupation (CWmP). B) Typical starvation-induced pupation (SiP). Modified from Nijhout (2008) [15].
