# Supplementary figures and images for: Insights into How Longicorn Beetle Larvae Determine the Timing of Metamorphosis: Starvation-Induced Mechanism Revisited
Source: PLoS One. 2016 Jul 7;11(7):e0158831. doi: 10.1371/journal.pone.0158831 (PMC4936689; doi:10.1371/journal.pone.0158831)

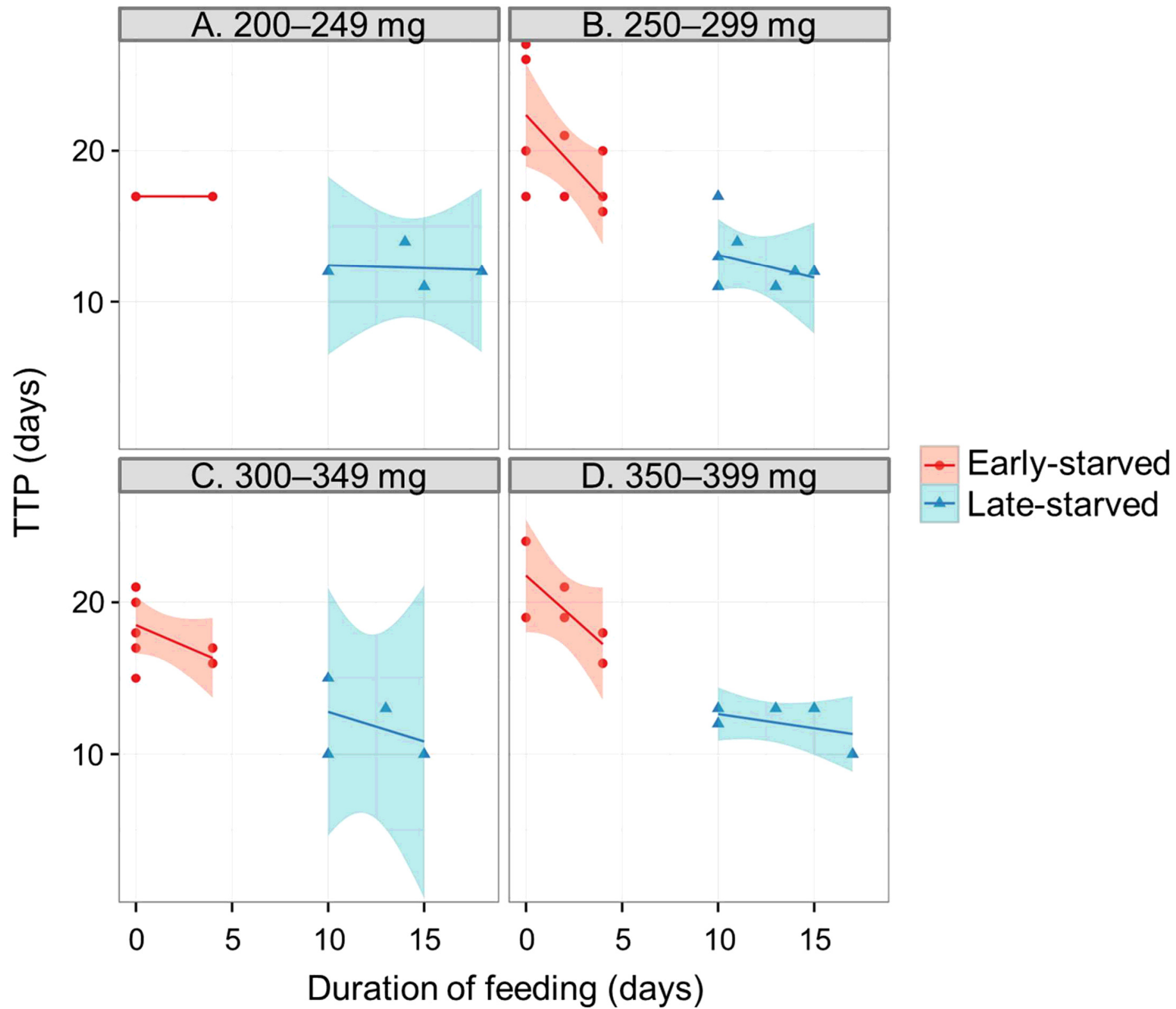

Supplement: S1 Fig — Samples of Fig 3A were divided into four groups (panels A–D) based on the initial weight in the 5th instar. Red and blue colors indicate the early- and late-starved groups, respectively. The 95% confidence intervals of the regression segments are indicated by shades. (PDF) [file pone.0158831.s001.pdf]

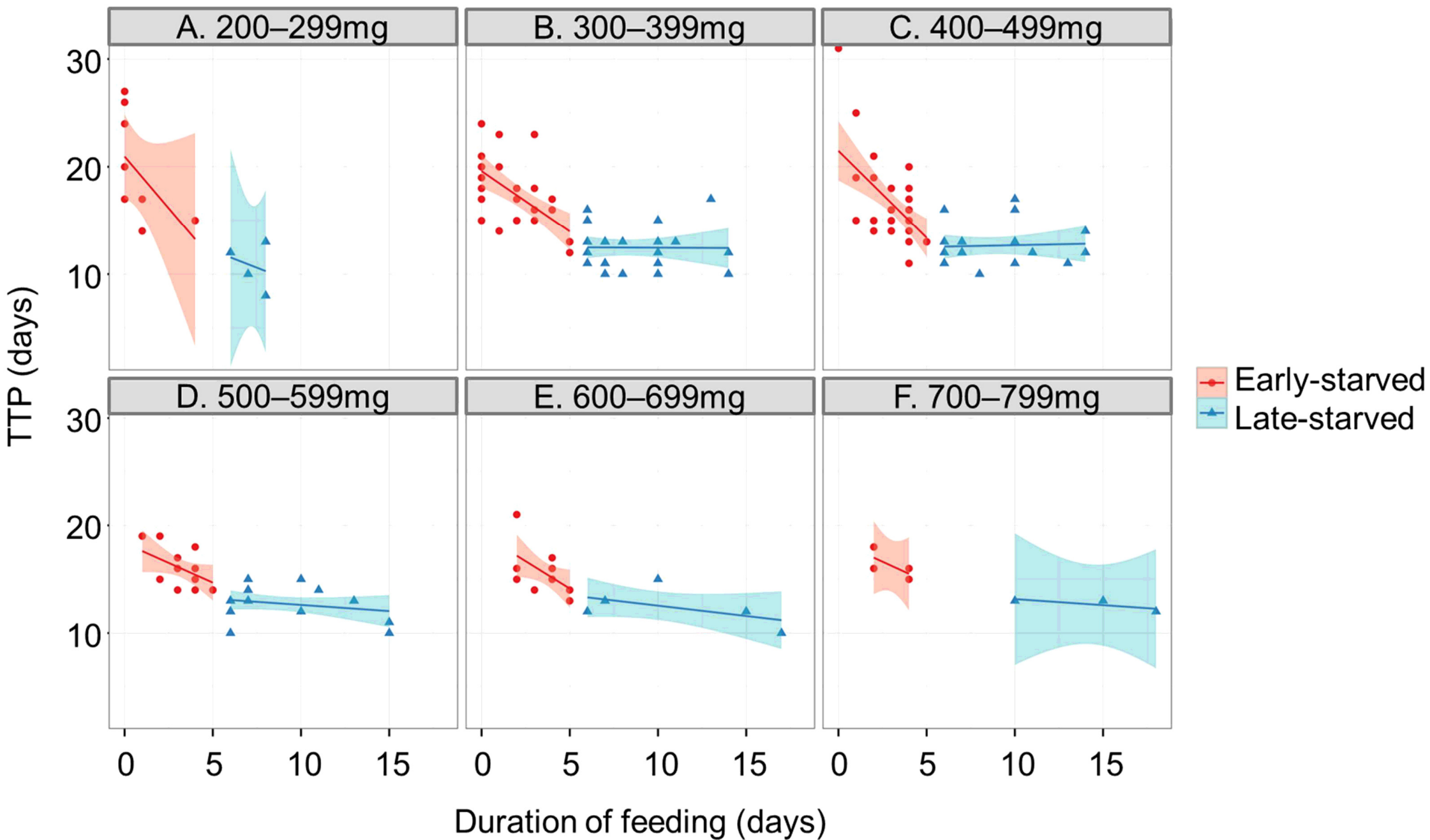

Supplement: S2 Fig — Samples of Fig 3A were divided into six groups (panels A–F) based on the peak weight in the 5th instar. Red and blue colors indicate the early- and late-starved groups, respectively. The 95% confidence intervals of the regression segments are indicated by shades. (PDF) [file pone.0158831.s002.pdf]
