# Supplementary material for: Insights into How Longicorn Beetle Larvae Determine the Timing of Metamorphosis: Starvation-Induced Mechanism Revisited
Source: PLoS One. 2016 Jul 7;11(7):e0158831. doi: 10.1371/journal.pone.0158831 (PMC4936689; doi:10.1371/journal.pone.0158831)
Supplement: S1 Table — (PDF) [file pone.0158831.s003.pdf]

S1 Table. Initial weight, duration of 5th instar, pupal weight, and pupal duration of *Psacothea hilaris*, larvae of which were starved early or late in the 5th instar

Regimen *	Initial weight at 5th instar (mg) §		Duration of 5th instar (days) §		Pupal weight (mg) §		Pupal duration (days) §	
	Mean (S.D.)	n	Mean (S.D.)	n	Mean (S.D.)	n	Mean (S.D.)	n
Continuously fed	333.7[a] (69.2)	39	26.0[c] (3.2)	59	427.7[c] (114.8)	59	12.2[a] (0.6)	26
Early-starved	330.8[a] (67.7)	28	19.4[a] (2.7)	111	257.1[a] (80.8)	111	11.7[a] (0.9)	23
Late-starved	305.0[a] (94.3)	36	21.5[b] (3.5)	85	306.9[b] (97.5)	85	11.7[a] (0.8)	26

* Early-starved: data for larvae fed for 0–5 days prior to starvation (0F-S to 5F-S in Fig. 2A) were combined. Late-starved: data for larvae fed for 6–18 days prior to starvation (6F-S to 18F-S in Fig. 2A) were combined.

§ Means in the same column with the same letter are not significantly different (Tukey test, $p < 0.05$).
