# Supplementary material for: Insights into How Longicorn Beetle Larvae Determine the Timing of Metamorphosis: Starvation-Induced Mechanism Revisited
Source: PLoS One. 2016 Jul 7;11(7):e0158831. doi: 10.1371/journal.pone.0158831 (PMC4936689; doi:10.1371/journal.pone.0158831)
Supplement: S2 Table — (PDF) [file pone.0158831.s004.pdf]

S2 Table. Results of refeeding experiments (late-starved). Weight at food deprivation and pupation rate in the 5th instar *P. hilaris* larvae fed for 10 days prior to starvation

Regimen *	Weight at food deprivation (mg) §		Pupation rate at the next ecdysis	
	Mean (S.D.)	n	%	n
10F-1S-F	530.2[a] (164.4)	9	82	11
10F-2S-F	568.0[a] (160.3)	6	75	8
10F-3S-F	472.1[a] (125.3)	20	100	20
10F-4S-F	610.8[a] (135.8)	10	100	10

* F and S indicate feeding and starvation, respectively. 10F-1S-F, for example, indicates that newly ecdysed 5th instar larvae were fed for 10 days, starved for 1 day, and fed again.

§ Means with the same letter are not significantly different (Tukey test, $p < 0.05$).
