# Supplementary material for: Insights into How Longicorn Beetle Larvae Determine the Timing of Metamorphosis: Starvation-Induced Mechanism Revisited
Source: PLoS One. 2016 Jul 7;11(7):e0158831. doi: 10.1371/journal.pone.0158831 (PMC4936689; doi:10.1371/journal.pone.0158831)
Supplement: S3 Table — (PDF) [file pone.0158831.s005.pdf]

S3 Table. Results of refeeding experiments (late-starved). Initial weight at 5th instar, pupal weight in weight-gain and weight-loss groups, and pupal duration in *P. hilaris*, larvae of which were fed for 10 days prior to starvation

Treatment *	Initial weight at 5th instar (mg) §		Pupal weight in weight-gain group (mg) §		Pupal weight in weight-loss group (mg) §		Pupal duration (day) §	
	Mean (S.D.)	n	Mean (S.D.)	n	Mean (S.D.)	n	Mean (S.D.)	n
10F-1S-F	322.1^{a} (80.3)	11	411.3^{a} (122.5)	9	- (-)	0	11.7^{a} (0.7)	9
10F-2S-F	320.8^{a} (97.0)	8	512.4^{a} (114.4)	5	249.0^{a} (-)	1	11.3^{a} (1.2)	6
10F-3S-F	305.1^{a} (76.0)	19	364.2^{a} (120.5)	6	292.7^{a} (95.3)	14	11.7^{a} (0.9)	20
10F-4S-F	356.4^{a} (111.0)	10	- (-)	0	387.9^{a} (102.7)	10	12.2^{a} (0.8)	10

* See the footnote to S2 Table.

§ Means in the same column with the same letter are not significantly different (Tukey test, $p < 0.05$).
