# Supplementary material for: Insights into How Longicorn Beetle Larvae Determine the Timing of Metamorphosis: Starvation-Induced Mechanism Revisited
Source: PLoS One. 2016 Jul 7;11(7):e0158831. doi: 10.1371/journal.pone.0158831 (PMC4936689; doi:10.1371/journal.pone.0158831)
Supplement: S4 Table — (PDF) [file pone.0158831.s006.pdf]

S4 Table. Results of refeeding experiments (early-starved). Weight at food deprivation and pupation rate in the 5th instar *P. hilaris* larvae fed for 4 days prior to starvation

Regimen *	Weight at food deprivation (mg) §		Pupation rate at the next ecdysis	
	Mean (S.D.)	n	%	n
4F-2S-F	477.4[a] (132.0)	10	100	10
4F-4S-F	450.0[a] (-)	1	100	1
4F-5S-F	464.7[a] (104.4)	3	100	3
4F-6S-F	496.6[a] (75.9)	7	100	7

* See the footnote to S2 Table.

§ Means with the same letter are not significantly different (Tukey test, $p < 0.05$).
