# Supplementary material for: Insights into How Longicorn Beetle Larvae Determine the Timing of Metamorphosis: Starvation-Induced Mechanism Revisited
Source: PLoS One. 2016 Jul 7;11(7):e0158831. doi: 10.1371/journal.pone.0158831 (PMC4936689; doi:10.1371/journal.pone.0158831)
Supplement: S5 Table — (PDF) [file pone.0158831.s007.pdf]

S5 Table. Results of refeeding experiments (early-starved). Initial weight at 5th instar, pupal weight in weight-gain and weight-loss groups, and pupal duration in *P. hilaris*, larvae of which were fed for 4 days prior to starvation

Regimen *	Initial weight at 5th instar (mg) §		Pupal weight in weight-gain group (mg) §		Pupal weight in weight-loss group (mg) §		Pupal duration (days) §	
	Mean (S.D.)	n	Mean (S.D.)	n	Mean (S.D.)	n	Mean (S.D.)	n
4F-2S-F	351.8[a] (102.0)	10	389.3[a] (147.9)	10	- (-)	0	11.7[a] (0.8)	10
4F-4S-F	358.0[a] (-)	1	454.0[a] (-)	1	- (-)	0	13.0[a] (-)	1
4F-5S-F	306.5[a] (29.0)	2	374.0[a] (91.9)	2	201.0[a] (-)	1	11.3[a] (1.2)	3
4F-6S-F	373.1[a] (56.7)	7	- (-)	0	303.0[a] (53.0)	7	11.4[a] (0.5)	7

* See the footnote to S2 Table.

§ Means in the same column with the same letter are not significantly different (Tukey test, $p < 0.05$).
